# On-Line Compensation for Micromilling of High-Aspect-Ratio Straight Thin Walls

**DOI:** 10.3390/mi12060603

**Published:** 2021-05-23

**Authors:** Yang Li, Xiang Cheng, Siying Ling, Guangming Zheng

**Affiliations:** 1 School of Mechanical Engineering, Shandong University of Technology, Zibo 255000, China; liyang0918@163.com (Y.L.); zhengguangming@sdut.edu.cn (G.Z.); 2 School of Mechanical Engineering, Dalian University of Technology, Dalian 116024, China; lingsy@dlut.edu.cn

**Keywords:** thin wall, deformation, dimensional error, cutting force measurement, cutting parameter compensation

## Abstract

In order to improve the machining quality and reduce the dimensional errors of micro high-aspect-ratio straight thin walls, the on-line cutting parameter compensation device has been introduced and corresponding micromilling processes have been investigated. Layered milling strategies for the micromilling of thin walls have been modeled and simulated for thin walls with different thicknesses based on the finite element method. The radial cutting parameters compensation method is adopted to compensate the thin wall deformation by raising the radial cutting parameters since the thin wall deformation make the actual radial cutting parameters smaller than nominal ones. The experimental results show that the dimensional errors of the thin wall have been significantly reduced after the radial cutting parameter compensation. The average relative dimensional error is reduced from 6.9% to 2.0%. Moreover, the fabricated thin walls keep good shape formation. The reduction of the thin wall dimensional error shows that the simulation results are reliable, which has important guiding significance for the improvement of thin wall machining quality, especially the improvement of dimensional accuracy. The experimental results show that the developed device and the machining strategy can effectively improve the micromilling quality of thin walls.

## 1. Introduction

The large deformation of thin walls with high aspect ratio finishing by micromilling is a challenging research topic. The serious elastic deformation recovery after the processing results in the size, shape, and position error of the thin wall workpiece [1]. The stiffness of thin wall parts decreases gradually from the bottom to the top. Therefore, the deformation at the top of the thin wall is greater than that at the bottom when the same cutting parameters are used in the machining process. The final processed thin wall parts would form a situation where the thickness at the top of the thin wall is greater than that at the bottom. However, this phenomenon would be significantly reduced if layered milling strategies are carried out. The residual material is hard to remove by a milling cutter in the cutting process due to machining deformations, which require repeated finishing, leading to low processing efficiency and possible repositioning errors. Therefore, it is a hot research topic of deformation compensation according to the predicted machining deformation in the machining process.

Many scholars have conducted a lot of studies on the mechanism of thin wall deformation and cutting parameter compensation for thin walls. Lim et al. proposed a method for compensating milling errors with ball-end milling cutters by using a controlled surface strategy. By this method, the machining errors caused by cutters were predicted using a surface generation model without the need for actual machining experiments. In order to adapt to different conditions of surface errors, error sensitive functions were defined when generating control surfaces [2]. Lim et al. studied the high-precision milling deformation of turbine blades with complex curved surfaces, and analyzed the influence of machining paths and milling parameters on the milling deformation [3]. Cho et al. proposed an error compensation system based on OMM (on-machine measurement) and introduced the concepts of error band and error deviation to compensate the machining errors caused by tool deformation. Then, the neural network algorithm was used to obtain the optimal cutting conditions to reduce the machining error by iterating the measured data [4,5]. Ratchev et al. used two ways of single-level cycle and multi-level cycle to compensate the tool path based on the prediction of machining deformation. The single-level cycle only compensated the tool path once without considering the coupling effect between the change of tool point and machining deformation. In the multi-level cycle method, iterative calculation was carried out between the modified tool point, the cutting force model and the machining deformation until the machining surface error meets the given accuracy requirements [6]. Chen et al. aimed at the accumulation of layering errors in cutting, started from the error model, compensated and corrected the machining trajectory of each layer, and compared the results of single-layer compensation and non-compensation to verify the machining accuracy of layering compensation [7]. Song et al. proposed a prediction model of cutting error and cutting force considering the coupling influence of tool, workpiece deformation and cutting force. The elastoplastic mechanical energy method was used to solve the deformation, and the validity of this method was verified by experiments [8]. Cao et al. established a numerical control milling deformation prediction model based on ABAQUS software (Version number: 2016, SIMULIA, Johnston, RI, USA). By comparing the model with the experimental results, they found that the model was not much different from the actual situation [9]. Bolar et al. established a three-dimensional milling simulation model for thin wall parts by using computer simulation technology. The model mainly analyzed the influence of milling force on the processing deformation [10]. Huang et al. aimed at the deformation problem of impeller five-axis side milling, used OMM technology to measure and reconstruct the machined surface, and modified the tool path with iterative compensation algorithm to reduce the machining error [11]. Wang et al. studied the relationship between the wall thickness of the parts and the milling force and the influence degree on the milling deformation for the size of thin wall parts. At the same time, the machining deformation in a certain time was calculated by the distribution of the milling force in the contact parts between the cutter and the workpiece [12]. Ratchev et al. made a certain analysis of the influence factors of common machining deformation and conducted a study on the compensation of stratified errors in milling. In addition, the algorithm based on the principle that different machining paths will produce different cutting forces finds out the dynamic balance relationship between the two paths, thus improving the accuracy of thin wall size [13]. Hou et al. aimed at the problem of cutting deformation in the process of thin wall parts machining, a learning control method on machining error compensation was proposed. Based on elastic deformation theory, the nonlinear function relationship between machining error and nominal cutting depth was established, and a general model of machining error compensation for thin wall parts was constructed by using the compensation idea, that is the calculation method of nominal cutting depth in the next cutting. The model realized the off-line learning of compensation coefficient and accurate control of machining error by using the feedback principle. Finally, the effectiveness of the model was verified by peripheral milling experiments [14].

In the above literatures, a large number of studies have been carried out on the deformation of thin wall parts in milling, and a variety of cutting parameter compensation methods for the deformation of thin wall parts have been put forward to continuously improve the processing quality of thin wall parts. However, the mentioned cutting parameter compensations are basically off-line optimization, and there are very few on-online compensations. Consequently, there are still serious limitations in improving the thin wall machining quality. Even on-line optimization is still requires changing the original tool path and generating a new machining program, resulting in relatively low efficiency. Therefore, a device for cutting force measurement and cutting parameter compensation is developed in this study. By comparing the cutting force measured in the thin wall machining process with the critical cutting force identified in the finite element simulation, the cutting parameter to be compensated is determined. Then, the cutting parameters are compensated on-line by the device.

## 2. Thin Wall Deformation Mechanism and Compensation Device

### 2.1. Thin Wall Deformation Mechanism

**Figure 1:**
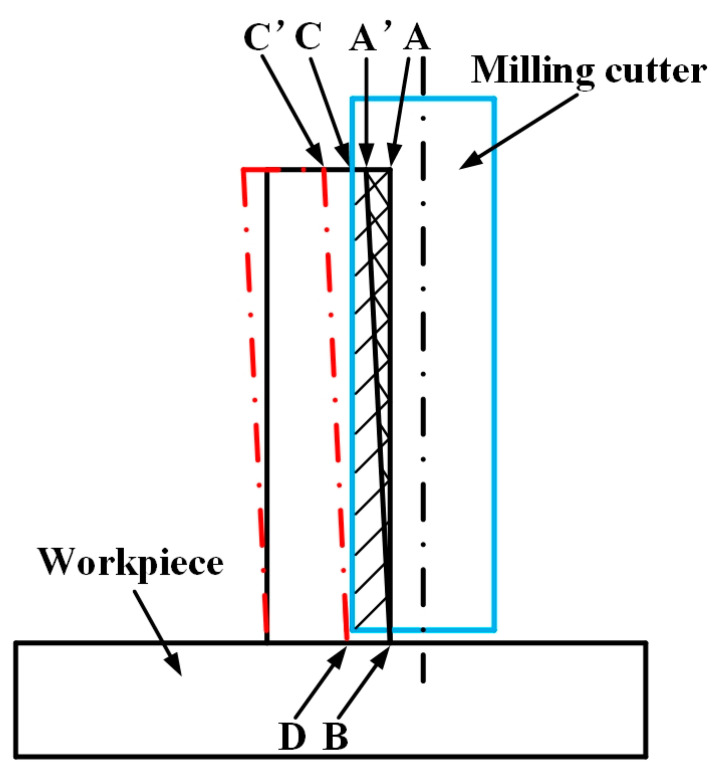
Error in thin wall machining.

The reason of machining error is shown in Figure 1 [10]. The materials of shadow part ABCD should be removed in theory when the end milling cutter is used to mill thin walls. But thin walls produce elastic deformation due to cutting forces, resulting in that two points A and C move to A’ and C’, respectively. Therefore, only the A’BCD part materials were removed by milling cutter. The elastic deformation of thin wall parts recovered after milling cutter leaves thin wall parts, and some residual C’CD materials are not removed, resulting in the wall thickness processing error. Consequently, the thickness at the top of the thin wall is greater than the thickness at the bottom of the thin wall. Therefore, the machining error of thin wall parts is mainly the fact that the cutter cannot remove all the materials due to the machining deformation of thin walls. If the deformation of the thin wall can be identified, the residual material can be removed by means of CNC machining compensations. Therefore, the key to solve the machining error of thin wall parts is to obtain the deformation values of the workpiece under the specific machining conditions.

### 2.2. Cutting Force Measurement and Cutting Parameter Compensation Device

The machining quality can be improved and the dimensional errors can be reduced by optimizing the tool path to compensate the cutting parameters [6]. However, it is necessary to modify the original tool path if considering the tool path compensation, which seriously affects the machining efficiency. Therefore, a device for cutting force measurement and cutting parameter compensation is developed in this study. It can measure the radial cutting force perpendicular to the thin wall that generated in the thin wall cutting process. Then, the radial cutting parameter is compensated according to the value of the cutting force.

**Figure 2:**
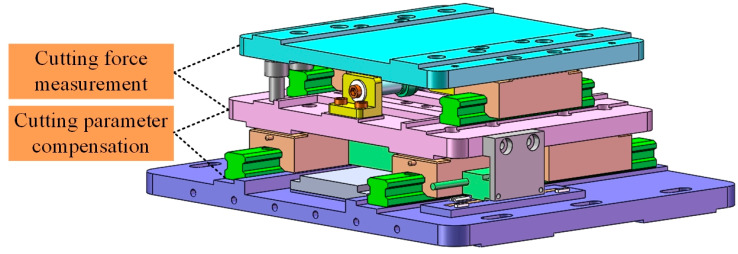
The designed cutting force measurement and cutting parameter compensation device.

Figure 2 shows the three-dimensional structure diagram of the designed cutting force measurement and cutting parameter compensation device. It is mainly composed of two parts, the cutting force measurement part and the cutting parameter compensation part. The cutting force measurement part is composed of a piezoelectric sensor, two guide rails and sliders. The resolution of the piezoelectric sensor (Model: 6023, ARIZON, Changzhou, China) is 0.001 N and the comprehensive accuracy is 0.005 N. The cutting parameter compensation part is composed of a linear motor (Model: AJM50, Akribis, Shanghai, China), two guide rails and sliders, and a grating ruler (Model: RGH41, RENISHAW, London, England). The resolution of the motion system is 0.1 μm.

**Figure 3:**
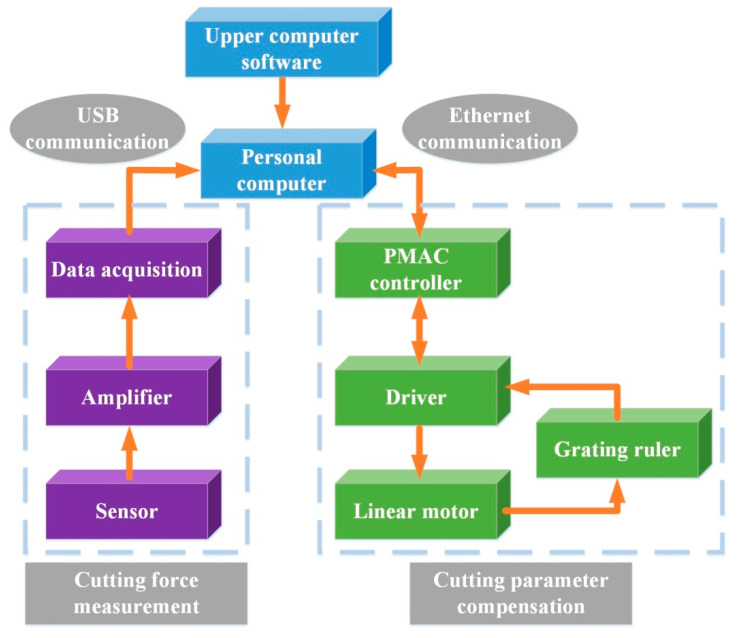
Control system diagram of the proposed device.

Figure 3 shown the control system diagram of the proposed device, which is also mainly divided into two parts. One is the force analog signal outputted by the force sensor being amplified and collected. The signal is transmitted to the computer after A/D conversion from the USB port and processed by the upper computer software to get the final measured cutting force value. Another is to realize the servo control of the linear motor for the compensations.

The upper computer software sends the motion instruction to the PMAC (Program Multiple Axis Controller, Delta Tau Data System; Chatsworth, CA, USA) controller through the ethernet port. The linear motor is used to realize the desired movement through the driver and the compensation is realized.

**Figure 4:**
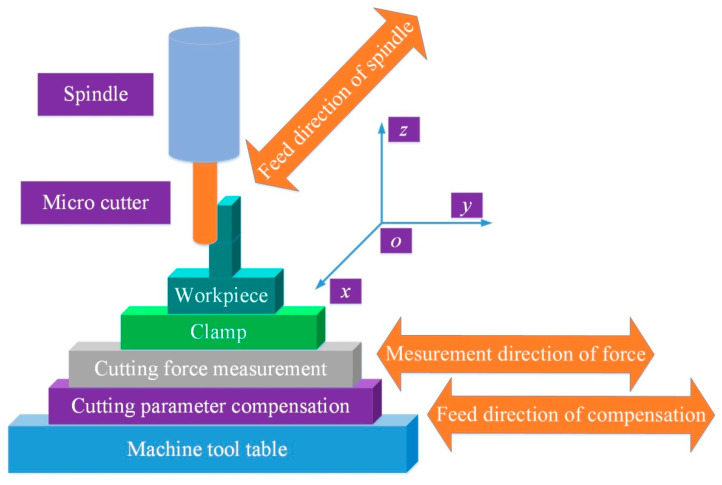
Working principle diagram of cutting force measurement and cutting parameter compensation device.

The working principle of the proposed device is shown in Figure 4. The spindle is fed in the direction parallel to the thin wall, and the cutting force measurement system is used to measure the radial cutting force perpendicular to the thin wall direction in the thin wall cutting process. After reading and processing the cutting force, the upper computer outputs the command to PMAC controller. The cutting parameter compensation system can realize the linear motion perpendicular to the thin wall direction, so as to realize the purpose of compensating the radial cutting parameter.

## 3. Finite Element Simulation

Due to the cutting force cannot directly reflect the size of the thin wall deformation, the finite element model is created for the simulation of H59 Brass material to conduct three-dimensional thin wall cutting simulations. The value of radial cutting parameter compensation for each layer of thin wall is identified by simulation. Then, the corresponding critical cutting force is identified to determine whether the radial cutting parameter needs to be compensated in the cutting process.

### 3.1. Simulation Model

**Figure 5:**
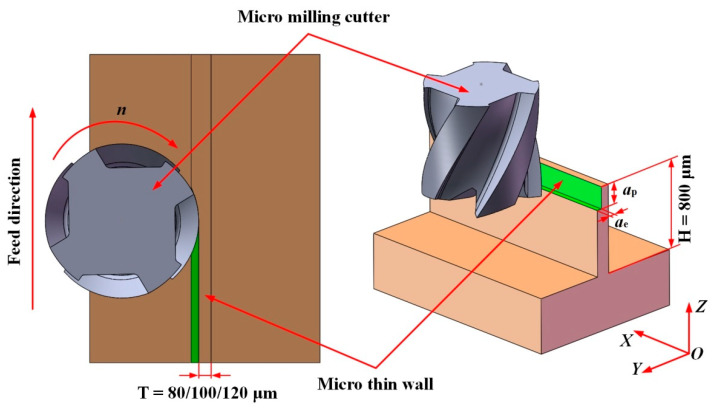
3D model of micro milling cutter and thin wall workpiece.

According to the general characteristics of end milling of thin wall parts, the three-dimensional models of four-tooth micro milling cutter and thin wall workpiece are established by the software SolidWorks (Version number: 2014, Dassault Systemes, Waltham, MA, USA), as shown in Figure 5. In Figure 5, H is the height of the thin wall, T is the thickness of the thin wall, *a*_e_ is the radial depth of cut, *a*_p_ is the axial depth of cut, and *n* is the spindle rotation speed.

Based on the finite element software Deform-3D (Version number: V11.0, Science Forming Technology Corporation, Columbus, OH, USA), the thin wall cutting model has been created for the simulation employing the classical Johnson-Cook constitutive material model. The geometric model of the cutter has been simplified to improve the simulation speed. The precutting preparation is conducted to the workpiece model and the assembled final element model. The reasonable meshing can ensure the simulation quality and reduce the simulation time. The tetrahedral mesh type is applied, and the local grids are refined with the size of 1 μm for cutting edges and the cutting zone in the workpiece. It can simultaneously guarantee the simulation precision, shorten the remeshing time and improve simulation speed. Furthermore, the adaptive meshing method has been applied and the mesh size can be refined according to the real-time calculation requirements. The bottom surface of the workpiece is completely fixed as a boundary condition, and the cutter feeds along the X-axis. The properties of the micro milling cutter and the material properties of the workpiece are shown in Table 1 and Table 2, respectively. The material H59 brass can be obtained conveniently from the market and its datasheet is available in the software Deform-3D.

**Figure 6:**
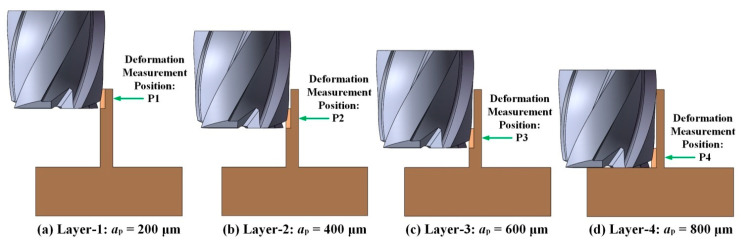
Thin wall deformation measurement by layered milling. (**a**) Layer-1; (**b**) Layer-2; (**c**) Layer-3; (**d**) Layer-4.

Considering that the stiffness of thin wall parts decreases from the bottom to the top, layered simulation is carried out for axial cutting to study the relationship between deformation amount and the number of axial layers, as shown in Figure 6 [15]. Three kinds of H59 Brass thin wall parts with the height of 800 μm and thickness of 80 μm, 100 μm, and 120 μm are simulated respectively under the same cutting parameters using the established finite element simulation model, and the change law of cutting force and deformation in the process of milling is analyzed.

### 3.2. Cutting Parameters Selection

The feed per tooth is directly related to the phenomenon of minimum cutting thickness, and has a great influence on the micro milling force and machining quality due to the size effect in the micro milling process. Therefore, the feed per tooth is an important parameter in the micro milling process. In this study, the main purpose is to study the relationship between milling force and deformation of the thin wall. Considering the size effect in the microcutting process, the nonlinear force increase due to ploughing should be avoided. Therefore, the feed per tooth is greater than the minimum undeformed chip thickness. At the same time, excessive feed per tooth values are not suitable to be selected, otherwise it will make the cutting force increase, leading to larger deformation of thin walls. Therefore, the thin wall cutting parameters are determined as shown in Table 3 based on previous studies [16,17,18,19,20,21].

### 3.3. Simulation Result and Analysis

**Figure 7:**
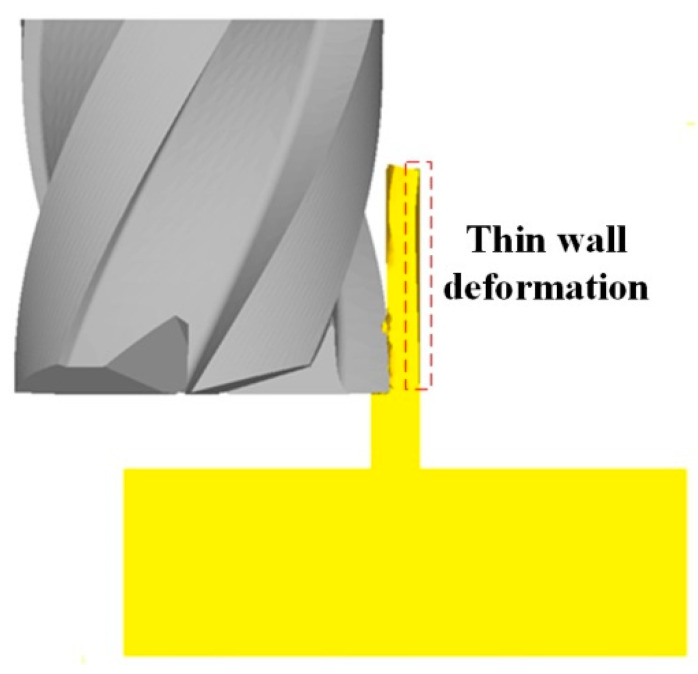
Thin wall deformation in simulation process.

**Figure 8:**
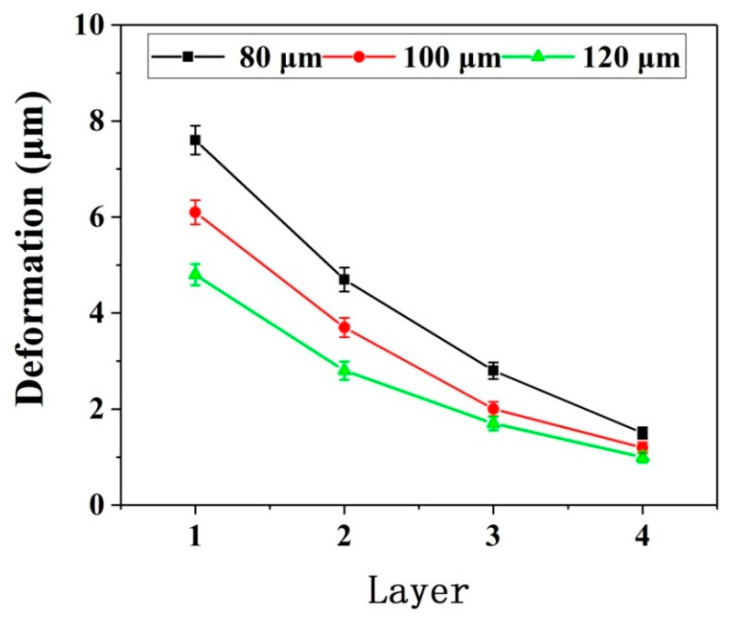
Thin wall deformation.

**Figure 9:**
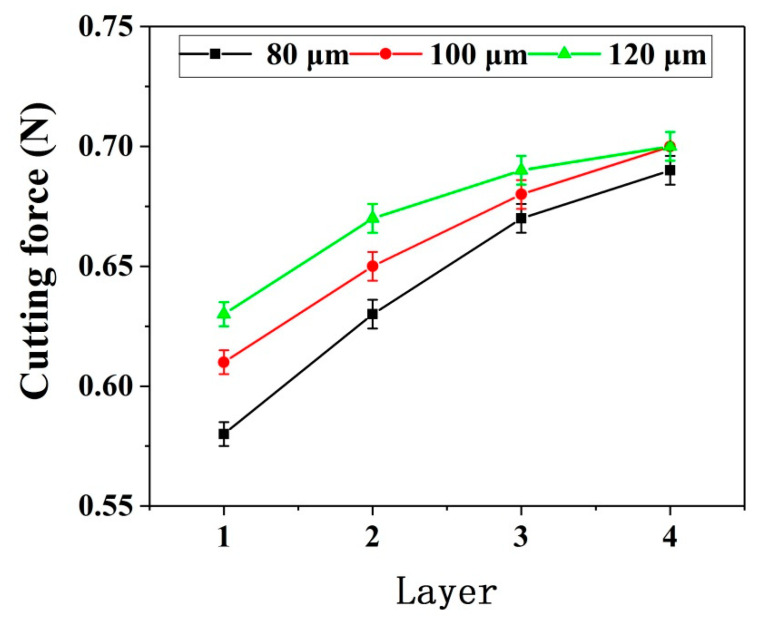
Thin wall cutting force.

Figure 7 shows the deformation of the thin wall in the simulation process. Figure 8 and Figure 9 show the deformation diagram and cutting force diagram of each layer of three thin walls with different thicknesses in layered milling.

It can be seen from Figure 8, the deformation of the thin wall decreases with the increase of the number of axial milling layers, indicating that the stiffness of the bottom of the thin wall is greater than that of the top of the thin wall. The ability of the bottom of the thin wall to resist deformation is stronger. Moreover, the thicker the thin wall, the more resistant it is to deformation. As be seen from Figure 9, the thin wall cutting force increases with the increase of the number of layers. The increase of cutting force is due to the continuous increase of effective radial cutting parameters, which indicates the continuous decrease of deformations. For the same number of layers and the same process conditions, the thicker the thin wall, the greater the cutting force. There is a negative correlation between the changing trend of cutting force and the changing trend of deformation.

### 3.4. Simulation for Radial Cutting Parameter Compensation

In order to reduce the dimensional errors of thin wall caused by machining deformation, the method of compensating radial cutting parameter is adopted to reduce the dimensional errors of thin walls. Since each compensation may lead to the increase of new deformations, it is necessary to carry out multiple compensations until the new deformation meets certain requirements. In this study, the first radial compensation value is set as the thin wall deformation value simulated, and the second compensation value is set as the thin wall added deformation after the first compensation. If the added deformation is less than 1 μm, the compensation is stopped. If the added deformation is greater than 1 μm, the compensation is continued until the added deformation is less than 1 μm.

The layered compensation for a thin wall with a thickness of 80 μm is carried out and analyzed. In the first layer, the average peak force reaches 0.73 N after three times compensation, the added deformation is 0.5 μm (less than 1 μm), and the total of radial cutting parameter compensation is 11.4 μm. In the second layer, the average peak force reaches 0.71 N after two times compensation, the added deformation is 0.9 μm (less than 1 μm), and the total of radial cutting parameter compensation is 6.6 μm. In the third layer, the average peak force reaches 0.72 N after two times compensation, the added deformation is 0.5 μm (less than 1 μm), and the total of radial cutting parameter compensation is 3.8 μm. In the fourth layer, the average peak force reaches 0.71 N after one time compensation, the added deformation is 0.7 μm (less than 1 μm), and the total of radial cutting parameter compensation is 1.5 μm.

Based on the above simulation results and analysis method, the total value of radial cutting parameter compensation of each layer and the nominal cutting parameter is taken as the actual radial cutting parameter for each layer, and the cutting force generated by the actual radial cutting parameter is taken as the critical cutting force for each layer in the thin wall milling experiment. The radial cutting parameter compensation value makes the cutting force reach the critical cutting force, and therein the compensation stops. Table 4, Table 5 and Table 6 summarize the important parameters of the thin wall simulation process with the thickness of 80 μm, 100 μm, and 120 μm, respectively.

## 4. Experiment

In order to verify the reliability of the simulation result and verify whether the thin wall dimensional errors are reduced after the cutting parameter compensation, a comparative experiment is conducted using the proposed device. The experiments are divided into two groups: the first group is the conventional thin wall micromilling experiment without cutting parameter compensation, and the second group is the compensation one.

### 4.1. Contrast Experiment Setup

**Figure 10:**
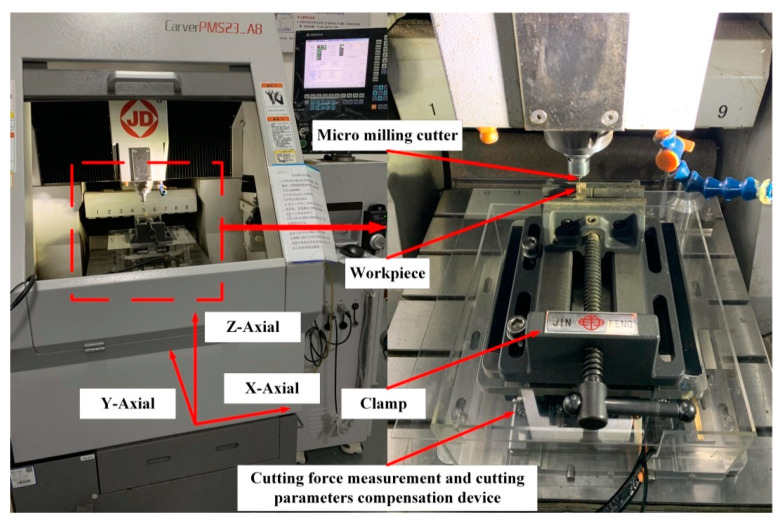
Experiment setup.

The CNC micromilling machine tool CarverPMS23_A8 (Beijing Jingdiao, Beijing, China) is selected to conduct the contrast experiments, as shown in Figure 10. The maximum rotation speed of spindle is 36,000 rpm. The cutting force measurement and cutting parameter compensation device is fixed on the worktable of the machine tool. The workpiece is fixed on the proposed device through the clamp. The cutter, workpiece, and cutting parameters used in the experiment are consistent with those in the simulation.

### 4.2. Results and Analyses

**Figure 11:**
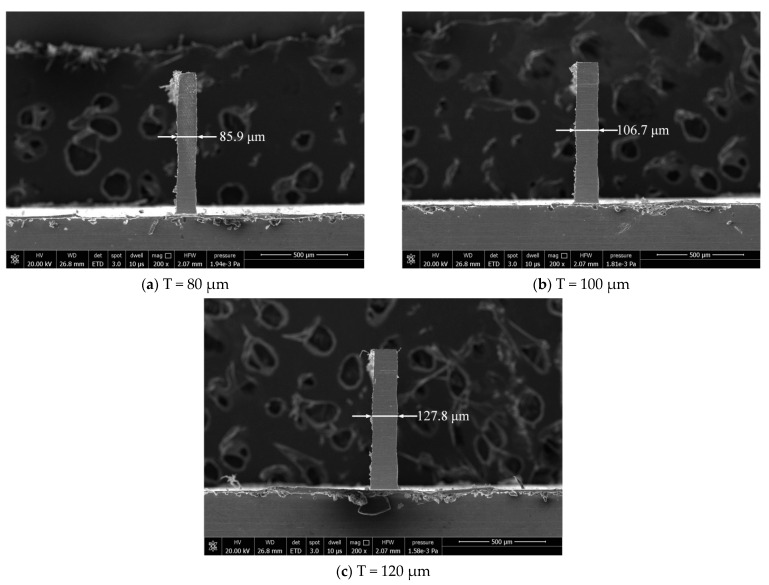
Results without radial cutting parameter compensation.

**Figure 12:**
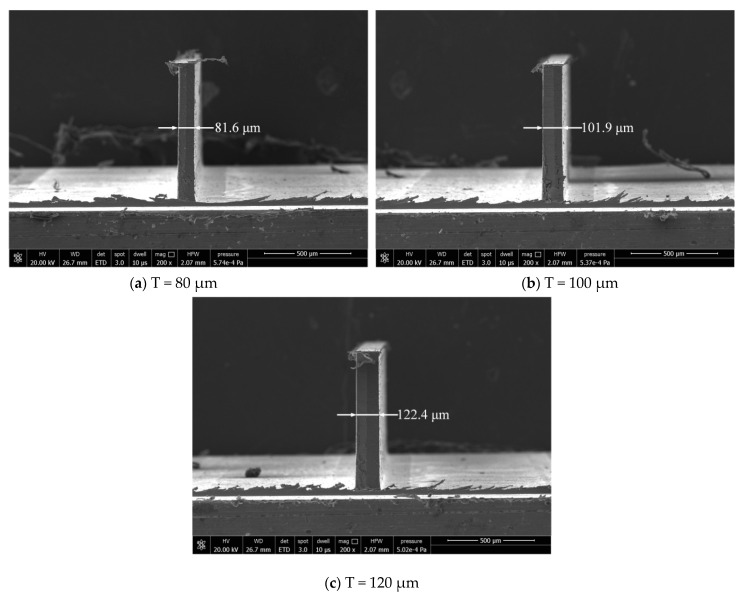
Results with radial cutting parameter compensation.

The dimensional error has some difference between the actually machined thickness and the ideal one of the thin wall. The actual thicknesses of thin walls are measured by scanning electron microscope (Model: Quanta 250, FEI, OR, USA) after milling. Thin wall thickness can be evaluated by measuring 4 thicknesses along the wall, Figure 11 and Figure 12 show the thin wall thickness measurement results without and with compensations, respectively. The standard deviations of the measured values can be calculated by Equation (1). Table 7 shows the thin wall relative dimensional errors results.

Relative dimensional errors = [(Actual thin wall thickness − Nominal thin wall thickness)/Nominal thin wall thickness] × 100% (1)

It can be seen from Figure 11 that the thickness of the thin wall without compensation is not uniform. But from Figure 12, the thickness of the thin wall after radial cutting parameter compensation is relatively uniform. The experiment results show that the dimensional errors of the thin walls have been significantly reduced. The average relative error has been reduced from 6.9% to 2.0%. The machining quality has a relatively obvious improvement in dimensional accuracy after the cutting parameter compensation. It is undeniable that the size of the thin wall still has a certain error.

**Figure 13:**
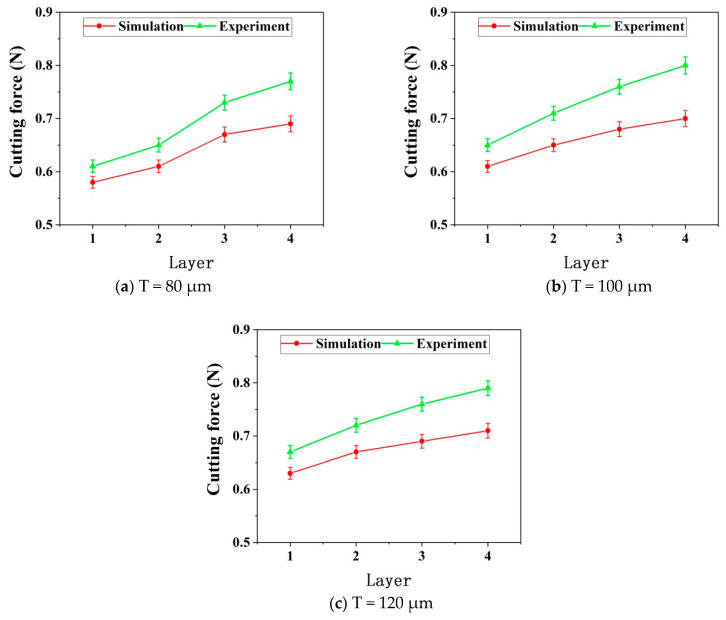
Cutting forces without radial cutting parameter compensation.

As can be seen from Figure 13, both the simulated cutting forces and the experimental cutting forces of the three thin walls with different thicknesses increase with the increase of the number of axial layers. Meanwhile, with the increase of the number of layers, the error between the simulated cutting force and the experimental one gradually increases. Possible reasons are discussed as follows.

After the first layer is cut, the elastic deformation of the thin wall is recovered. Part of the material is not removed due to the deformation. After the second layer is cut, since the thin wall stiffness of the second layer is greater than that of the first layer under the same process conditions, the deformation of the second layer will be less than that of the first layer in the cutting process. Therefore, the residual material of the first layer will be partially removed at this time. Similarly, when the third layer is cut, the residual material of the first layer and the second layer will be partially removed too. Furthermore, when the fourth layer is cutting, the residual material of the previous three layers will be partially removed. Therefore, in this cutting situation, the material removal amount from top to bottom of each layer is increasing.

However, the model of each layer is different in the simulation process. When the next layer is simulated, the material of the upper layer is set to be completely removed. Therefore, the error between the simulated cutting force and the experimental one is constantly increasing with the increase of the number of cutting layers due to the above reasons.

As shown in Figure 11, the dimension errors of the three thin walls don’t appear in the situation where the thickness at the top of the thin wall is significantly larger than that at the bottom of the thin wall, as shown in Figure 1. The reason for this is that, in the process of layered cutting, the tool constantly removes the uncut part of the material on the upper layer, and the final thin wall will not appear obvious shape error on the whole, but there will be a certain error on the dimension. Axial laminated milling can improve the shape accuracy of thin wall parts, but it is difficult to reduce the dimensional errors.

Since the actual cutting experiments need to remove a small part of residual material of the upper layer, so the actual compensations have not reached the values in the simulation when the measured cutting force values have reached the critical cutting force values. That is an important reason for the dimensional errors of thin walls in the final analysis.

### 4.3. Optimization Experiment Setup

In order to determine whether the added deformation value of 1 μm is a reasonable compensation threshold, further optimization experiments are carried out. According to the simulation results, it is found that after the last compensation, the added deformation value is within the interval of 0.5 μm to 0.9 μm. Therefore, further optimization is carried out according to this result, and the optimization experiments are divided into two groups.

In the first group, the added deformation in the range of 0.5 μm to 0.9 μm is further compensated to obtain a small added deformation, making the added deformation fall within the range of 0.1 μm to 0.5 μm. In the second group, the compensation value of radial cutting parameter is modified to obtain a large amount of added deformation, so that the added deformation falls within the range of 0.9 μm to 1.3 μm. Then, experiments are carried out respectively to compare and analyze the machining quality of thin walls. All the optimization experimental parameters are summarized as shown in Table 8.

### 4.4. Optimization Results and Analyses

**Figure 14:**
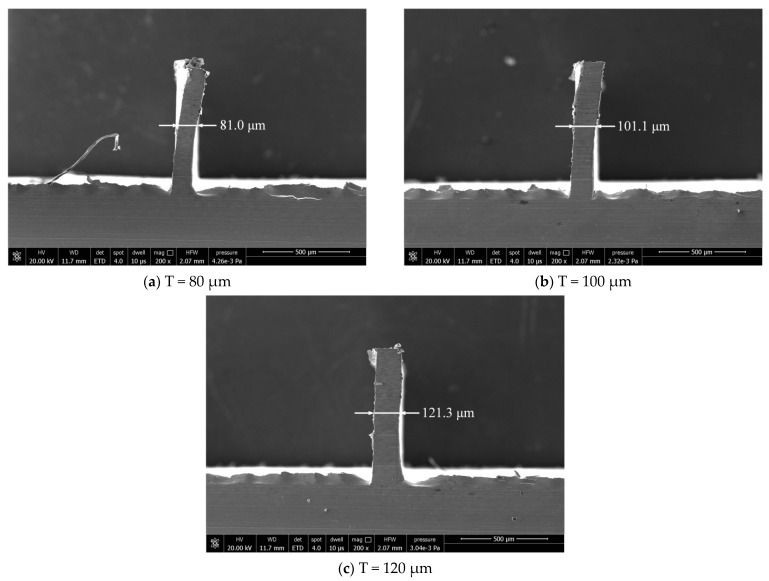
Thin wall measurement results of the first group optimization experiments.

**Figure 15:**
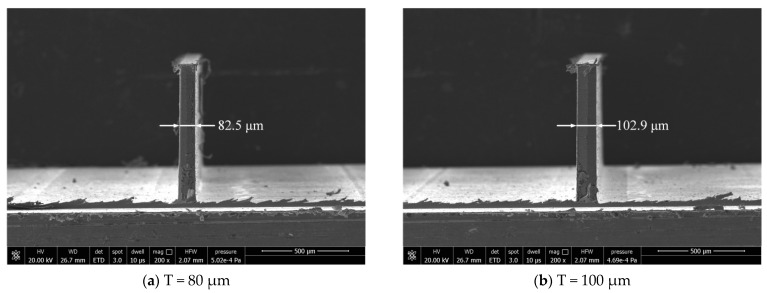

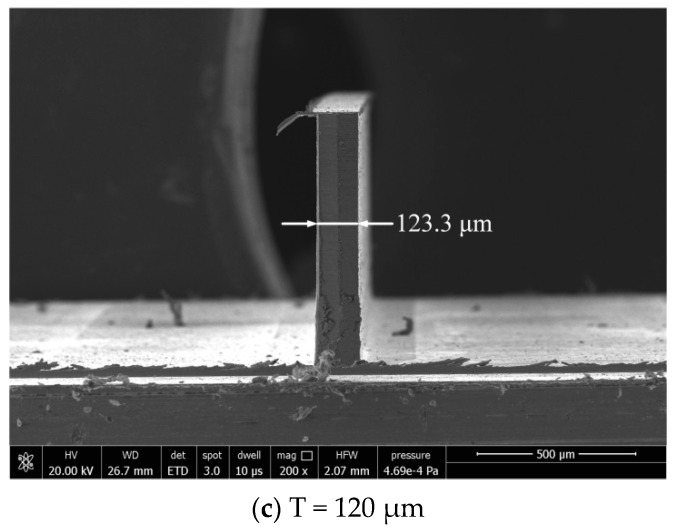
Thin wall measurement results of the second group optimization experiments.

The optimization experiment results of the two groups are shown in Figure 14 and Figure 15 respectively.

According to the results of the two groups of optimization experiments shown in Figure 14 and Figure 15, the thin wall shape accuracy is evaluated. The evaluation criteria for shape accuracy are that the thin wall has not bending deformation and the thin wall thickness is uniform. The evaluation results are shown in Table 9. In the first group of optimization experiments, different degrees of bending deformation appear on the top of the two thin walls with the thickness of 80 μm and 100 μm, which might be caused by the excessive radial cutting values. The shape accuracy is relatively poor. The results of the second group are consistent with good shape accuracies. According to the results of the two groups of optimization experiments shown in Figure 14 and Figure 15, the relative dimensional errors are evaluated by Equation (1), and the results are shown in Table 10. The relative dimensional errors of the first group are less than that before optimization. The relative dimensional errors of the second group are greater than that before optimization.

By comparing the above two evaluation results, it is found that the smaller the added deformation after multiple radial cutting parameter compensation, the smaller the dimensional errors of the thin wall. However, due to the radial cutting parameter at the top of the thin wall is too large, the shape accuracy of the thin wall is poor. The larger the added deformation is, the larger the dimensional errors of the thin wall will be, but the higher the shape accuracy of the thin wall becomes. Therefore, it is considered that the machining quality of the thin wall will be better when the compensation value of radial cutting parameters makes the added deformation within the range of 0.5–0.9 μm considering the shape accuracy and dimensional errors of the thin wall together.

## 5. Conclusions

Based on finite element simulations and micromilling experiments, the proposed device for radial compensations in micromilling of thin walls has been evaluated and micromilling strategies have been created successfully. The simulation results show that the deformation at the top of the thin wall is greater than that at the bottom with the same cutting parameters. Namely, the stiffness at the top of the thin wall is less than that at the bottom. For finishing thin walls with the same heights by the same process conditions, the thicker the thin wall is, the greater the stiffness and the smaller the deformation will be. Experimental results show that the simulation results are reliable, which has important guiding significance for the actual machining and experimentation with the thin wall. The contrast experiments show that the dimensional errors of the thin wall are significantly reduced after the radial cutting parameter compensation. The average relative error has been reduced from 6.9% to 2.0%. The dimensional accuracy of the thin wall has been greatly improved and the shape accuracy of the thin wall has been guaranteed compared with that without compensations. According to the experimental results, the final processed thin wall parts wouldn’t form a situation where the top thickness is obviously greater than that at the bottom. The reason is that layered milling strategies significantly reduce this phenomenon. Therefore, layered milling strategies are necessary for raising the machining quality of thin walls with high aspect ratios.

## Figures and Tables

**Table 1:** Micro milling cutter properties.

Diameter	Spiral Angle	Cutting Edges	Cutting Edge Radius	Matrix	Coating	Tool Length
1 mm	35°	4	5 μm	Carbide	TiAlN	50 mm

**Table 2:** Workpiece material properties.

Material	Elastic Modulus (N/mm^2^)	Shear Elastic Modulus (N/mm^2^)	Poisson Ratio	Thermal Conductivity (W/(m·K))	Specific Heat (J/(kg·K))	Density (Kg/m^3^)
H59 Brass	100,000	37,593	0.33	110	390	8500

**Table 3:** Selection of simulation parameters.

No.	T (μm)	H (μm)	*a*_e_ (μm)	*a*_p_ (μm)	*f*_z_ (μm/z)	*n* (rpm)
1	80					
2	100	800	50	200	1.6	20,000
3	120					

**Table 4:** The simulation results for the thin wall of 80 μm thickness.

Compensation Step		Layer			
		1	2	3	4
No compensation	Average peak force (N)	0.58	0.61	0.67	0.69
	Deformation (μm)	7.6	4.7	2.8	1.5
First compensation	Radial compensation (μm)	7.6	4.7	2.8	1.5
	Average peak force (N)	0.67	0.69	0.70	0.71
	Added deformation (μm)	2.6	1.9	1.2	0.7
Second compensation	Radial compensation (μm)	2.6	1.9	1.2	
	Average peak force (N)	0.70	0.71	0.72	
	Added deformation (μm)	1.2	0.9	0.5	
Third compensation	Radial compensation (μm)	1.2			
	Average peak force (N)	0.73			
	Added deformation (μm)	0.5			

**Table 5:** The simulation results for the thin wall of 100 μm thickness.

Compensation Step		Layer			
		1	2	3	4
No compensation	Average peak force (N)	0.61	0.65	0.68	0.70
	Deformation (μm)	6.1	3.7	2.0	1.2
First compensation	Radial compensation (μm)	6.1	3.7	2.0	1.2
	Average peak force (N)	0.68	0.70	0.70	0.71
	Added deformation (μm)	2.1	1.3	0.9	0.5
Second compensation	Radial compensation (μm)	2.1	1.3		
	Average peak force (N)	0.70	0.72		
	Added deformation (μm)	1.1	0.7		
Third compensation	Radial compensation (μm)	1.1			
	Average peak force (N)	0.73			
	Added deformation (μm)	0.5			

**Table 6:** The simulation results for the thin wall of 120 μm thickness.

Compensation Step		Layer			
		1	2	3	4
No compensation	Average peak force (N)	0.63	0.67	0.69	0.71
	Deformation (μm)	4.8	2.8	1.7	1.0
First compensation	Radial compensation (μm)	4.8	2.8	1.7	1.0
	Average peak force (N)	0.69	0.70	0.71	0.72
	Added deformation (μm)	1.7	1.0	0.7	0.5
Second compensation	Radial compensation (μm)	1.7	1.0		
	Average peak force (N)	0.72	0.73		
	Added deformation (μm)	0.8	0.5		

**Table 7:** Comparison of relative dimensional errors.

No.	Thin Wall Thickness (μm)	Thin Wall Thickness without Compensation (μm)	Relative Dimensional Errors	Thin Wall Thickness with Compensation (μm)	Relative Dimensional Errors
1	80	85.9	7.4%	81.6	2.0%
2	100	106.7	6.7%	101.9	1.9%
3	120	127.8	6.5%	122.4	2.0%
Average	100	107.2	6.9%	102.0	2.0%

**Table 8:** Optimization experiment parameters.

Thin Wall Thickness (μm)	Added Deformation Interval (μm)	Add Deformation (μm)/Radial Compensation (μm)/Critical Cutting Force (N)			
		1st Layer	2nd Layer	3rd Layer	4th Layer
	(0.1, 0.5)	0.1/11.9/0.74	0.2/7.5/0.73	0.1/4.3/0.73	0.1/2.2/0.72
80	(0.5, 0.9)	0.5/11.4/0.73	0.9/6.6/0.71	0.5/3.8/0.72	0.7/1.5/0.71
	(0.9, 1.3)	1.2/10.2/0.70	1.2/3.9/0.66	1.2/2.6/0.70	↑
	(0.1, 0.5)	0.1/9.8/0.75	0.2/5.7/0.74	0.2/2.9/0.72	0.1/1.7/0.72
100	(0.5, 0.9)	0.5/9.3/0.73	0.7/5.0/0.72	0.9/2.0/0.70	0.5/1.2/0.71
	(0.9, 1.3)	1.1/8.2/0.70	1.3/3.7/0.70	↑	↑
	(0.1, 0.5)	0.2/7.3/0.74	0.1/4.3/0.74	0.1/2.4/0.72	0.1/1.5/0.73
120	(0.5, 0.9)	0.8/6.5/0.72	0.5/3.8/0.73	0.7/1.7/0.71	0.5/1.0/0.72
	(0.9, 1.3)	1.2/3.9/0.66	1.0/2.8/0.70	↑	↑

**Table 9:** Shape accuracy of thin wall.

Added Deformation Interval (μm)	Thin Wall Thickness (μm)		
	80	100	120
(0.1, 0.5)	★	★★	★★
(0.5, 0.9)	★★★	★★★	★★★
(0.9, 1.3)	★★★	★★★	★★★

The more number of “★”, the better the shape accuracy of the thin wall.

**Table 10:** Thin wall relative dimensional errors.

Added Deformation Interval (μm)	Thin Wall Thickness (μm)		
	80	100	120
(0.1, 0.5)	1.0%	1.1%	1.2%
(0.5, 0.9)	2.0%	1.9%	2.0%
(0.9, 1.3)	3.0%	2.9%	3.1%

## Data Availability

All data are given in the paper. No further data sets were used.
